# Racial Disparities in Access to Dental Care: A Secondary Analysis of the California Health Interview Survey 2023

**DOI:** 10.31661/gmj.v13iSP1.3731

**Published:** 2024-12-29

**Authors:** Khadim Hussain Hamid, Behzad Vosooghinezhad, Omid Tavakol, Mehrnoosh Khoshnevisan, Mahnaz Gholami, Mina Khayamzadeh, Moslem Karimzadeh

**Affiliations:** ^1^ Department of Periodontics, Mashhad University of Medical Science, Mashhad, Iran; ^2^ Dentistry Department, European University, Tibilisi, Georgia; ^3^ Private Practice Periodontologist, Shiraz, Iran; ^4^ Department of Pediatric Dentistry, School of Dentistry, Ahvaz Jundishapur University of Medical Science, Ahvaz, Iran; ^5^ Bushehr University of Medical Science, Periodontics Department, Bushehr, Iran; ^6^ Department of Oral and Maxillofacial Medicine, School of Dentistry, Tehran University of Medical Sciences, International Campus, Tehran, Iran; ^7^ Islamic Azad University Tehran Medical Sciences, Tehran, Iran

**Keywords:** Racial Disparities, Dental Care, Access to Care, Health Inequities, Oral Health, California Health Interview Survey

## Abstract

Background: Racial disparities in dental health care access have been a persistent issue in the United States. This study aims to investigate the association between racial disparities and access to dental care. Materials and Methods: This study conducted a secondary data analysis of the CHIS 2023 Adult Survey, which included a sample of 21671 individuals. The survey collected information on demographic characteristics, health behaviors, and dental health outcomes, including access to dental care. Ordinal regression analysis was performed to examine the association between racial disparities and dental service outcomes, controlling for demographic and socioeconomic factors. Results: The study found significant differences in demographic characteristics among racial groups. The 2023 CHIS analyzed 21,671 individuals across various racial groups, revealing significant variations in age distribution, with White individuals being the oldest and “Other single race” the youngest. Whites also had the highest median income and dental insurance coverage. An ordered logistic regression adjusted for age, gender, educational level, income last month, current smoking status, and dental insurance showed that being White was associated with a significantly higher number of dental visits (coefficient = 0.14, P-value = 0.005), while other racial groups did not show significant associations. Conclusion: This study suggests that racial disparities exist in dental service outcomes in California, with certain racial and ethnic groups being less likely to access dental care. The findings show the need to address these disparities and improve access to dental care for marginalized populations.

## Introduction

Oral health is an integral component of overall health and well-being, playing a critical role in the quality of life of individuals and communities worldwide. The mouth is a complex ecosystem that harbors a diverse array of microorganisms, with an estimated 700 species of bacteria, viruses, fungi, and protozoa coexisting in a delicate balance [1]. This intricate ecosystem is susceptible to disruptions, which can lead to a range of oral health issues, including dental caries, periodontal disease, and oral cancer, among others. These conditions not only compromise the oral health of individuals but also have far-reaching consequences for overall health, including increased risk of cardiovascular disease, diabetes, and respiratory infections [2][3]. Regular dental visits are a crucial aspect of preventive oral healthcare, enabling early detection and treatment of oral diseases, and promoting overall health and well-being. Despite the importance of oral health, many individuals fail to receive regular dental care, leading to a significant burden of untreated oral diseases, such as tooth decay, periodontal disease, and oral cancer [4][5]. In the United States alone, it is estimated that over 40% of adults have not visited a dentist in the past year, and disparities in dental care access and utilization exist across various sociodemographic groups, including low-income populations, racial and ethnic minorities, and rural residents [6][7]. In the United States, significant racial and ethnic disparities in dental care persist, resulting in unequal access to oral health services, poorer dental outcomes, and increased burden of oral diseases among marginalized populations. Despite advances in dental technology and widespread recognition of the importance of oral health, racial and ethnic minorities continue to experience substantial barriers to dental care [8]. According to the Centers for Disease Control and Prevention (CDC), in 2019, non-Hispanic black and Hispanic adults were more likely to have untreated cavities, missing teeth, and periodontal disease compared to non-Hispanic white adults [9]. Moreover, American Indian/Alaska Native populations have the highest rates of dental caries and tooth loss among all racial and ethnic groups [9]. These disparities are not solely the result of individual-level factors, such as oral health behaviors or socioeconomic status. Rather, they are deeply rooted in systemic and structural issues, including inadequate access to dental insurance, limited availability of culturally competent dental providers, and inequitable distribution of dental resources [10]. So, this study aims to investigate the association between racial disparities and access to dental care.

## Materials and Methods

We conducted a secondary data analysis of the California Health Interview Survey (CHIS) 2023 Adult Survey [11], which is part of a series of methodological reports describing the 2023 CHIS. The CHIS is a collaborative project between the University of California, Los Angeles (UCLA) Center for Health Policy Research, the California Department of Public Health, and the Department of Health Care Services. Social Science Research Solutions (SSRS) was responsible for data collection.

The CHIS sample design was optimized to meet two primary objectives:

Provide estimates for large- and medium-sized counties in California, as well as groups of the smallest counties (based on population size).

Provide statewide estimates for California’s overall population, its major racial and ethnic groups, and several racial and ethnic subgroups.

The CHIS sample is representative of California’s non-institutionalized population living in households. The sample design involved a multi-stage process, where households were first selected, and then an adult within each household was sampled. If there were children and/or adolescents in the household, one child and/or one adolescent was eligible for sampling.

Data collection for CHIS 2023 was conducted via telephone survey, with SSRS responsible for data collection. The survey instrument was designed to collect extensive information on health status, health conditions, health-related behaviors, health insurance coverage, access to health care services, and other health-related issues.

Data processing procedures for CHIS 2023 involved several steps, including data cleaning, editing, and weighting. The data were processed to ensure accuracy, completeness, and consistency. Weighting and variance estimation procedures were also applied to ensure that the sample was representative of the California population.

## Statistical Analyses

The selected variables from the California Health Interview Survey (CHIS) 2023 include demographic characteristics such as age, gender, educational level, and income, as well as health behaviors including current smoking status. Additionally, the variables also encompass access to care, including dental insurance and times visited dentist in last year.

times visited dentist in last year was considered as outcome. Cross tabulation by Chi-square test was performed in univariable analysis to see differences between different racial groups. A ordinal regression was performed to evaluate existence of any racial disparities in access to dental healthcare by adjusting for significant variables of univariable analysis. All were performed in STATA/MP17 with P-value of lower than 0.05 being considered as statistically significant.

## Results

**Figure-1:**
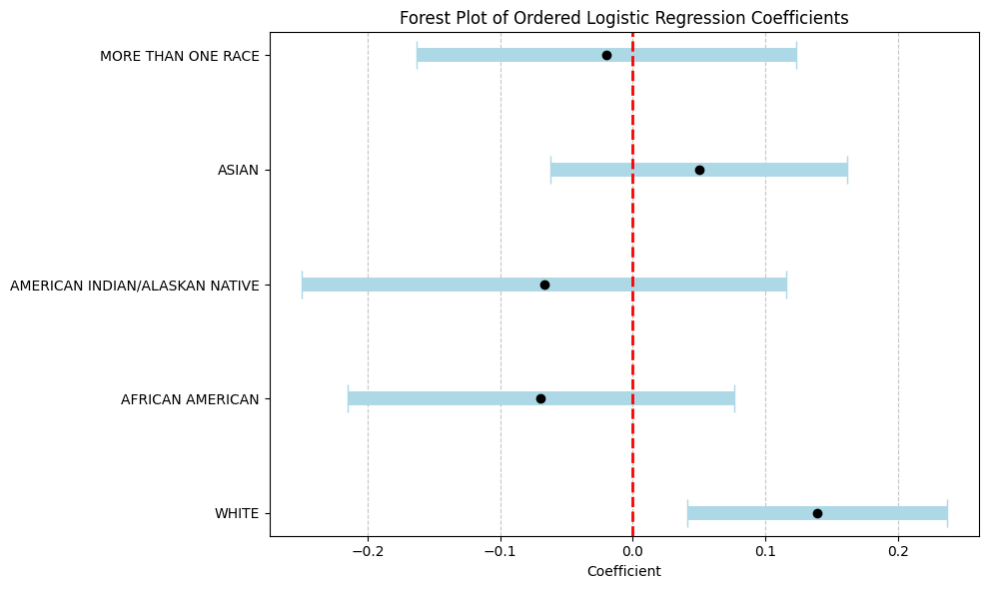
Figure 1. Ordinal Regression Results for Racial Disparities in Dental Service Outcomes

**Table:** Table1. Characteristics of subjects of CHIS 2023

		Other single race	American Indian/Alaska	Asian	African american	White	More than one race	P
	N	1,615	529	3,407	1,064	13,977	1,079	
	18-25	152(9.41%)	51(9.64%)	207(6.08%)	53(4.98%)	568(4.06%)	83(7.69%)	<0.001
	26-29	71(4.4%)	21(3.97%)	142(4.17%)	27(2.54%)	439(3.14%)	49(4.54%)	
	30-34	113(7%)	50(9.45%)	246(7.22%)	64(6.02%)	791(5.66%)	101(9.36%)	
	35-39	131(8.11%)	49(9.26%)	273(8.01%)	62(5.83%)	924(6.61%)	96(8.9%)	
	40-44	161(9.97%)	54(10.21%)	278(8.16%)	68(6.39%)	1048(7.5%)	103(9.55%)	
	45-49	161(9.97%)	41(7.75%)	319(9.36%)	97(9.12%)	995(7.12%)	99(9.18%)	
	50-54	181(11.21%)	52(9.83%)	355(10.42%)	105(9.87%)	1197(8.56%)	100(9.27%)	
Age, n(%)	55-59	156(9.66%)	44(8.32%)	339(9.95%)	110(10.34%)	1377(9.85%)	98(9.08%)	
	60-64	173(10.71%)	65(12.29%)	319(9.36%)	142(13.35%)	1659(11.87%)	107(9.92%)	
	65-69	138(8.54%)	44(8.32%)	340(9.98%)	136(12.78%)	1511(10.81%)	90(8.34%)	
	70-74	101(6.25%)	23(4.35%)	271(7.95%)	89(8.36%)	1419(10.15%)	77(7.14%)	
	75-79	40(2.48%)	23(4.35%)	156(4.58%)	59(5.55%)	1050(7.51%)	45(4.17%)	
	80-84	22(1.36%)	10(1.89%)	94(2.76%)	31(2.91%)	575(4.11%)	22(2.04%)	
	85+	15(0.93%)	2(0.38%)	68(2%)	21(1.97%)	424(3.03%)	9(0.83%)	
Gender, n(%)	Male	650(40.25%)	220(41.59%)	1665(48.87%)	392(36.84%)	5936(42.47%)	416(38.55%)	<0.001
	Female	965(59.75%)	309(58.41%)	1742(51.13%)	672(63.16%)	8041(57.53%)	663(61.45%)	
Educational level, n(%)	less than high school	302(18.7%)	42(7.94%)	91(2.67%)	39(3.67%)	460(3.29%)	33(3.06%)	<0.001
	high school	350(21.67%)	88(16.64%)	219(6.43%)	151(14.19%)	1734(12.41%)	135(12.51%)	
	Some college	518(32.07%)	215(40.64%)	525(15.41%)	388(36.47%)	3879(27.75%)	380(35.22%)	
	College degree	445(27.55%)	184(34.78%)	2572(75.49%)	486(45.68%)	7904(56.55%)	531(49.21%)	
Income, median (IQR)	1600(4543)	2000(5101)	3000(9334)	1300(6001)	1500(6701)	2000(6201)	<0.001
Current smoker	84(5.2%)	31(5.86%)	135(3.96%)	115(10.81%)	732(5.24%)	75(6.95%)	<0.001
Dental insurance	1098(67.99%)	380(71.83%)	2557(75.05%)	876(82.33%)	10119(72.4%)	830(76.92%)	<0.001
times visited dentist in last 12 months, median (IQR)	2(4)	2(4)	3(2)	2(4)	3(2)	3(4)	<0.001

**IQR:** interquartile range

The data represents a survey of 21,671 individuals, categorized by racial groups (American Indian/Alaska Native, Asian, African American, White, and More Than One Race). According to the CHIS data, the demographic distribution shows that White individuals make up the largest percentage at 64.50%, followed by Asian at 15.72%, while those identifying with more than one race, African American, American Indian/Alaska, and Other single race constitute the remaining population with percentages ranging from 2.44% to 7.45%.

The Table-1 provides a detailed demographic breakdown of the subjects in the 2023 California Health Interview Survey (CHIS), focusing on age distribution across different racial groups. The data reveals significant variations in age distribution among the racial categories. For instance, the "Other single race" group has a higher proportion of younger individuals, with 9.41% in the 18-25 age range, compared to the "White" group, which has only 4.06% in the same age range. Conversely, the "White" group has a larger proportion of older individuals, with 11.87% in the 60-64 age range and 10.81% in the 65-69 age range, indicating a more aged population. The "African American" group also shows a higher concentration of older individuals, with 12.78% in the 60-64 age range and 7.51% in the 75-79 age range. The "Asian" and "American Indian/Alaska Native" groups have relatively balanced age distributions, with no single age group having a notable presence of middle-aged individuals, such as 10.42% of the "Asian" group and 9.83% of the "American Indian/Alaska Native" group in the 50-54 age range. The "More than one race" group shows a relatively even distribution across most age ranges, with a slight concentration in the 30-34 and 50-54 age groups. The P-value of <0.001 indicates that these differences in age distribution across racial groups are statistically significant, suggesting that age and race are not independently distributed in the population surveyed. (Table-1).

We conducted an ordered logistic regression to analyze the number of dental visits in the last year, adjusting for age, gender, educational level, income last month, current smoking status, and dental insurance. The model converged after 3 iterations, with a final log likelihood of -34327.68. The number of observations was 21,671, and the likelihood ratio chi-squared test (LR chi2) was 1923.57 with a P-value of 0.0000, indicating a significant fit. The coefficients for race categories revealed that being White was associated with a significantly higher number of dental visits (coefficient = 0.14, P-value = 0.005) compared to the reference group. In contrast, being American Indian/Alaskan Native (coefficient = -0.07, P-value = 0.473), Asian (coefficient = 0.05, P-value = 0.381), African American (coefficient = -0.07, P-value = 0.351), and More than One Race (coefficient = -0.02, P-value = 0.787) did not show significant associations with the number of dental visits. These findings suggest that race may play a role in dental visit frequency, with White individuals being more likely to visit the dentist more frequently compared to other racial groups (Figure-1).

## Discussion

Our study investigated the association between racial disparities and access to dental care, revealing significant differences in demographic characteristics among racial groups, with White individuals being the oldest and having the highest median income and dental insurance coverage. In contrast, a study by Wehby et al. [12] found that racial and ethnic disparities in dental services use declined after Medicaid adult dental coverage expansions, with a reduction in disparities by 75% for non-Hispanic Black adults and 50% for Hispanic adults. However, another study [13] showed that children from all racial and ethnic groups experienced declines in receipt of dental visits during the COVID-19 pandemic, with prepandemic disparities in receipt of dental visits persisting for Black children and Asian children compared to White children. Furthermore, research by Shi et l. [14] indicated that Blacks and Hispanics were less likely to report difficulties in accessing medical care, dental care, and prescriptions compared to Whites, with these disparities occurring primarily among the uninsured and Medicaid insured. Additionally, a study by Liu [15] found that poor and minority children were less likely to receive preventive dental care, even when insurance status was considered.

The findings of our study are consistent with previous research [12][16][17][18], which have consistently shown that racial disparities exist in dental service outcomes, with certain racial and ethnic groups being less likely to access dental care. Specifically, our study found that being White was associated with a significantly higher number of dental visits, while other racial groups did not show significant associations. This is in line with the findings of other studies, which have reported that non-Hispanic Whites are more likely to receive annual dental examinations and have better access to dental care compared to other racial and ethnic groups [16][17][18]. Furthermore, our study highlights the need to address these disparities and improve access to dental care for marginalized populations, which is also emphasized in other research [17][18].

In contrast, a previous study [19] examined the role of dentist characteristics in income and racial/ethnic disparities in access to dental care, highlighting the importance of monitoring characteristics of dentists in addition to traditional measures of supply. Our study’s findings, which showed that being White was associated with a significantly higher number of dental visits, are consistent with the existing literature [19], suggesting that racial disparities exist in dental service outcomes in California, with certain racial and ethnic groups being less likely to access dental care.

The study’s limitations should be noted. The CHIS 2023 Adult Survey data may not be generalizable to other populations or geographic locations. Additionally, the survey’s reliance on self-reported data may introduce bias. Future studies should consider using multiple data sources and objective measures of dental health outcomes to validate these findings.

In conclusion, this study’s findings show the need to address racial disparities in dental health care access in California. The results are consistent with previous studies and underscore the importance of considering the role of socioeconomic factors in shaping dental health disparities. Future research should aim to explore the underlying causes of these disparities and develop targeted interventions to improve access to dental care for marginalized populations.

## Conflict of Interest

None declared.
